# Collaborative management measures of subsurface drainage and bio-organic fertilizer application for coastal sunflower (*Helianthus annuus L.*) based on TOPSIS entropy weight method

**DOI:** 10.1371/journal.pone.0318571

**Published:** 2025-04-09

**Authors:** Qinyuan Zhu, Jingnan Chen, Hanyi Rui, Yousef Alhaj Hamoud, Amal Mohamed AlGarawi, Mohammad K. Okla, Lin Zhu, Hiba Shaghaleh

**Affiliations:** 1 Nanjing Institute of Environmental Sciences, MEE, Nanjing, China; 2 College of Horticulture and Gardening, Fujian Agricultural Vocational and Technical College, Fuzhou, China; 3 College of Hydrology and Water Resources, Hohai University, Nanjing, China; 4 Botany and Microbiology Department, College of Science, King Saud University, Riyadh, Saudi Arabia; 5 College of Environment, Hohai University, Nanjing, China; Ardakan University, IRAN, ISLAMIC REPUBLIC OF

## Abstract

Soil salinization has become a global resource and ecological issue, and sunflower planting has had a good improvement effect on saline-alkali land. The study explores the collaborative management measures of subsurface drainage and bio-organic fertilization with high-yield, high-quality, and environmentally friendly sunflowers through experiments. We designed three subsurface pipe spacings (10, 15, and 20 m) and six methods of combined application of organic fertilizer (organic fertilizer nitrogen 100%, organic fertilizer nitrogen 75% + inorganic fertilizer nitrogen 25%, organic fertilizer nitrogen and inorganic fertilizer nitrogen each 50%, organic fertilizer nitrogen 25% + inorganic fertilizer nitrogen 75%, 100% inorganic fertilizer nitrogen, and no fertilizer treatment). Nine evaluation indexes were selected for the four aspects of yield increase, quality improvement, soil improvement, and emission reduction, and an index system was constructed. In the evaluation model, the TOPSIS entropy weight method was calculated to compare and select the most suitable growth method of subsurface drainage and bio-organic fertilizer application for sunflower growth in saline-alkali land. The results showed that the best treatment was 75% organic fertilizer nitrogen +  25% inorganic fertilizer nitrogen, and the best spacing for the subsurface drainage was 10 m. Under this treatment, the relative application progress reached 0.574, and the yield, oleic acid content, soil organic matter content, soil salt reduction efficiency, and N_2_O emissions were 2.93 t/ha, 21.73%, 2.21%, 37.62%, and 9.86 kg/ha, respectively.

## Introduction

Sunflower is a cash crop with tall plants, developed roots, high yield, large particles, good quality, and other favorable characteristics [1]. Sunflower is the fourth major oilseed crop worldwide [2]. It has been cultivated in multiple countries. According to statistics, the global planting area of sunflowers was about 27.36 million hm^2^ in 2019 [3]. China is a country that plants sunflowers, which are mainly distributed in the northeast, north, and northwest of China. The planting area of sunflowers is about 1 million hm^2^, and the total production remains between 2.4 and 2.6 million tons [4]. Compared with other crops, sunflowers have a strong tolerance to salt and alkali, barren land, and drought, with an especially strong tolerance to salt and alkali [5]. Studies have shown that when an area is planted with sunflowers, the soil surface salt content decreases, and soil fertility increases. Sunflower planting has a good improvement effect on saline-alkali land, and it is suitable for large-scale planting in coastal areas. Thus, it is one of the main crops used for the biological control of saline-alkali land [6].

Soil salinization has become a global resource and ecological issue. Saline alkali land is widely distributed in arid regions such as India and Pakistan, as well as coastal plains such as the Songnen Plain in China [7]. Currently, the methods to improve soil salinization focus on the following points: change the physical structure of soil by leveling the land and utilizing surface cover; apply chemical amendments to the soil, such as gypsum and sulfuric acid; adopt water conservancy engineering measures, such as desalination of drainage; apply organic fertilizers and plant salt-tolerant crops to reduce soil salinity [8,9].

Subsurface drainage was first developed in the UK and widely used in countries such as the US and Japan. The function of the subsurface has become increasingly diverse; at first, it was a single drainage, and later, it was water and salt drainage [10]. Subsurface drainage can effectively reduce soil salinity and control groundwater levels to prevent the return of salts [11]. Subsurface drainage eliminates excess water and salt in soil via large quota leaching, which can significantly reduce the EC value in salinized soil and improve the water use efficiency of crops, thereby increasing yield. Subsurface drainage has become an essential infrastructure in high-standard farmland construction projects and has been widely applied in arid and semi-arid areas in China with shallow saline and alkaline groundwater [12–14]. Bio-organic fertilizers are also one of the important measures to improve saline-alkali land. Bio-organic fertilizer has long fertilizer efficiency and low fertilizer loss rate; it can improve the yield and quality of crops [15], it can increase soil nutrients and organic matter, and have a positive impact on saline-alkali land [16]. Previous studies have shown that subsurface drainage and bio-organic fertilizer application can improve the soil structure in saline-alkali areas, increase the amount of soil organic matter in the topsoil, and accelerate the transformation of soil nitrogen from organic to mineral [17]. The combination of these two treatments has broad application prospects in saline-alkali land improvement [18]. China’s saline-alkali cultivated land amounts to 7.6 million hm^2^ [19], which is widely distributed in the Huanghuai Sea wheat area [20]. Due to the shortage of freshwater resources, population growth, and a decrease in the cultivated land area, the productivity of crops on saline-alkali land requires urgent improvement; such improvement would have great significance for the rational development and utilization of saline-alkali “reserve cultivated land” resources and in guaranteeing regional food security [21].

There have been many studies on saline-alkali soil improvement by means of subsurface drainage and bio-organic fertilizer application. However, there is little research on the coupling of the two measures, and there have been even fewer studies on optimal “saline-alkali soil/sunflower” management measures using different pipe spacings and different proportions of organic and inorganic fertilizer. This study compared different subsurface pipe spacings and bio-organic fertilizers in sunflower planting in saline-alkali soil and explored the effects of different methods on sunflower growth and environmental improvement, aiming to select the best mode and strive to improve the planting environment of crops in saline-alkali land.

As the largest agricultural reclamation area in China, the slip-mud reclamation area has the characteristics of a typical coastal agricultural area. The soil is dense and has high salinity, but its nutrient level is average. To develop modern agriculture, soil improvement is necessary to balance reserve cultivated land and protect the ecology. This study compared different pipe spacing and different application ratios of organic and inorganic fertilizers, choosing indicators from four aspects, including increasing yield, improving quality, improving soil, and reducing emissions, and then an index system was constructed. Through calculations based on the TOPSIS entropy weight method, a subsurface drainage and bio-organic fertilizer management model for sunflower growth in saline-alkali land was constructed. This model will provide a theoretical and practical foundation for sunflower planting in saline-alkali land.

## Materials and methods

### Experimental site

The experiment was carried out in the Tiaozi Mud reclamation area from June 8 to October 10, 2021. The Tiaozi Ni Reclamation Area is located in the coastal radiation sandbar of Dongtai City, Jiangsu Province, China(32°43’N ~ 32°53’N, 120°52’E ~ 120°58’E) (the experiment was permitted by the field owner Liu Qiang), and situated in the inner edge of the radiation sandbar. It was founded in 2014, and the reclaimed area covers 6096 hectares. It is an important arable land resource in China. The Tiaozi mud reclamation area belongs to the coastal facies sedimentary geomorphology unit, and this area belongs to the Lianlu mudflat, from Liangduo estuary to the north of Fangtang River Gate, which is a coastal sedimentary landform. A large amount of sediment carried by the Yangtze River, the ancient Yellow River, and the Huaihe River is deposited here, forming huge and loose deposits. The experimental site is located at the junction of a subtropical and a warm temperate region. It has a significant monsoon climate and abundant precipitation. Its temperature and humidity are 15.0°C and 81%, respectively. The soil condition of the experimental site is shown in Table 1.

**Table 1:** Soil condition of experimental site.

Soil type	Salt content (g kg^ − 1^)	Organic matter (%)	Soil available nitrogen (mg g^ − 1^)	Soil available phosphorus (mg g^ − 1^)	Soil available potassium (mg g^ − 1^)
Yellow-brown soil	4.2	2.1	114.6	8.8	110.7

### Experimental design

The research object of this study was sunflower (*Helianthus annuus L.*) plants. The growth period of the sunflowers was divided into the seedling stage (June 8 to July 21), budding stage (July 22 to August 15), full flowering stage (August 16 to August 30), and ripe picking stage (August 31 to October 10). Irrigation was conducted using municipal water (July 15, August 4, August 19, September 16). The respective irrigation amounts were 77mm, 83mm, 74mm, and 63mm. Throughout the experiment, the cumulative rainfall was recorded at 92mm, while the actual total water uptake by the sunflowers amounted to 389mm.

This experiment encompassed three different subsurface pipe spacings and six organic fertilizer application methods, resulting in a total of 3 (subsurface pipe spacings) x 6 (organic fertilizer application methods) =  18 treatment combinations. The experimental design details are outlined in Table 2. There were a total of 18 planting methods in this experiment, and each method was repeated three times. Apart from the variation in subsurface pipe spacings, all other field management practices were kept consistent. The concealed pipe was made of PVC, with 5 pipes laid for each planting method. The buried depth of the concealed pipe was 1.2m, with a concealed pipe ratio reduction of 0.1% and a density of 70 g/m^2^. Experimental fertilizers were urea, superphosphate, and potassium chloride, while organic fertilizer from Nanjing Mingzhu Fertilizer Co., Ltd was also employed. Controlled applications of P_2_O_5_, K_2_O, and N were maintained at rates of 120 kg/ha, 85 kg/ha, and 160 kg/ha, respectively, with contents measuring at levels of 2.3%, 1.2%, and 1.3%. Additionally, a central observation area measuring 5m x5m was established within each planting plot to facilitate enhanced monitoring of sunflower growth and development, alongside soil sampling along the cross-sections.

**Table 2:** Experimental design scheme (the organic fertilizer nitrogen is given in percent) .

Treatment	Pipe Spacing (m)	Combined Application of Organic Fertilizer
W_1_	10	100% organic fertilizer nitrogen
W_2_	10	75%organic fertilizer nitrogen + 25%inorganic fertilizer nitrogen
W_3_	10	50%organic fertilizer nitrogen + 50%inorganic fertilizer nitrogen
W_45_	10	25%organic fertilizer nitrogen + 75%inorganic fertilizer nitrogen
W_5_	10	100%inorganic fertilizer nitrogen
CK_1_	10	No nitrogen fertilizer
W_6_	15	100% organic fertilizer nitrogen
W_7_	15	75%organic fertilizer nitrogen + 25%inorganic fertilizer nitrogen
W_8_	15	50%organic fertilizer nitrogen + 50%inorganic fertilizer nitrogen
W_9_	15	25%organic fertilizer nitrogen + 75%inorganic fertilizer nitrogen
W_10_	15	100%inorganic fertilizer nitrogen
CK_2_	15	No nitrogen fertilizer
W_11_	20	100% organic fertilizer nitrogen
W_12_	20	75%organic fertilizer nitrogen + 25%inorganic fertilizer nitrogen
W_13_	20	50%organic fertilizer nitrogen + 50%inorganic fertilizer nitrogen
W_14_	20	25%organic fertilizer nitrogen + 75%inorganic fertilizer nitrogen
W_15_	20	100%inorganic fertilizer nitrogen
CK_3_	20	No nitrogen fertilizer

CK is a treatment without applying nitrogen fertilizer

### Determination indexes and calculation methods

#### Production increase index.

The sunflower yield was characterized by measuring the disc mass and 100 seeds at the maturity stage and converting these values to the yield per hm^2^ during data analysis.

#### Quality improvement indexes.

The indexes used to characterize the quality of the sunflowers included the palmitic acid (%), oleic acid (%), and linoleic acid (%) contents; determine the above indicators using gas chromatography [22,23].

#### Soil improvement indexes.

There are four indicators that characterize soil improvement, including soil available nitrogen content, available phosphorus content, organic matter content, and topsoil salt reduction efficiency.

Topsoil samples were collected in the middle of the sunflower ripening stage using the five-point method. The soil samples were air-dried, declutched, ground, and screened (0.15mm aperture) in the laboratory to determine the values of each index. The contents of available phosphorus were determined by means of the 0.5mol/LNaHCO_3_ extraction/molybdenum–antimonial resistance colorimetric method, available nitrogen was obtained through the alkali hydrolysis diffusion method, determination of soil organic matter using potassium dichromate sulfuric acid oxidation external heating method [24,25].

Soil samples were collected in layers, and their electrical conductivity was determined by means of air drying, grinding, sifting, leaching (soil-to-water ratio, 5:1), and filtration. The instrument for measuring conductivity is the DDSJ-308F (0.5 level) instrument (produced by Shanghai Leici Instrument Co., Ltd.). The conversion formula is as follows [26]:


S=4.634EC-0.626 (1)

where S is the total salt content, g/kg; EC is the conductivity value, ms/cm.

#### Emission reduction index.

N_2_O emissions were used as the emission reduction index. N_2_O gas was collected using a self-made PVC collection box a total of 11 times: on 3 consecutive days after sunflower sowing and each 15 days afterward. The N_2_O concentration was determined using a meteorological chromatograph. N_2_O emissions were calculated using the following formula [27]:


$$F=\rho \frac{V}{A}\frac{\Delta \text{c}}{\Delta \text{t}}\frac{273}{273+T}$$ (2)

where F is the amount of N_2_O emitted, μg/m^2^h; ρ is the density under N_2_O standard state, kg/m^3^; V is the volume of the collection box, m^3^; A is the sampling area, m^2^; ∆c/∆t is the change of N_2_O concentration time-dependent in the collection box; T is the average temperature of the collection box, °C.

An interpolation method was used to calculate the N_2_O emission flux on the unobserved dates between adjacent measurements, and the cumulative N_2_O emissions were obtained by means of summation. The formula for calculating the cumulative N_2_O emissions was as follows:


$$M=\sum \left(F_{N+1}+F_{N}\right)\times 0.5\times \left(F_{N+1}-t_{N}\right)\times 24$$ (3)

where M is the cumulative amount of N_2_O emitted, kg/ha; F is the amount of N_2_O emitted, μg/m^2^h, N is the number of samples; t is the number of days between adjacent measurements.

### Data analysis

Based on the research methods by Gao and Lian et al. [28,29], an indicator system was constructed. The indicators’ data were submitted to the SPSS17.0 software to calculate the significant differences. The improved TOPSIS method was applied to Deng Weihua [30].

## Results and analysis

### Index system construction

The construction of indicators takes into account the growth and development characteristics of sunflowers, the improvement of saline-alkali soil, and the impact on the environment. Nine indicators were selected in this study. The yield increase index is measured by the yield. The quality improvement indicators include palmitic acid content, oleic acid content, and linoleic acid content, which are important indicators reflecting the quality of sunflowers. Considering the impact of sunflowers on the soil environment and the improvement of saline-alkali soil, soil available nitrogen content, soil available phosphorus content, soil organic matter content, and topsoil salt reduction efficiency were set as soil improvement indicators. Considering the impact of sunflower cultivation on the atmospheric environment, N_2_O emissions were adopted as the emission reduction indicator. The index system and definitions are shown in Table 3, and the significance analysis of the indicator system is shown in Table 4.

**Table 3:** Index system for the subsurface drainage and bio-organic fertilizer management model for sunflower growth.

Criterion Layer	Index Level	Index Attribute
Yield increase	yield (t/hm^2^)	+
Quality improvement	palmitic acid content (%)	+
	oleic acid content (%)	+
	linoleic acid content (%)	+
Soil improvement	Soil available nitrogen content (mg/kg)	+
	Soil available phosphorus content (mg/kg)	+
	Soil organic matter content (%)	+
	Topsoil salt reduction efficiency (%)	+
Emission reduction	N_2_O emissions (kg/ha)	–

“ + “ is a positive indicator, meaning that a larger value is better, while” - “ is a negative indicator, meaning that a smaller value is better.

**Table 4:** Indicators under different treatments.

Treatment	yield (t/hm^2^)	palmitic acid content (%)	oleic acid content (%)	linoleic acid content (%)	Soil available nitrogen content (mg/kg)	Soil available phosphorus content (mg/kg)	Soil organic matter content (%)	Topsoil salt reduction efficiency (%)	N_2_O emissions (kg/ha)
W_1_	2.74 ± 0.11d	5.75 ± 0.06e	22.12 ± 0.3a	67.73 ± 0.4a	134.93 ± 15.1a	9.21 ± 0.15a	2.23 ± 0.11a	42.62 ± 3.36ab	8.85 ± 1.33c
W_2_	2.93 ± 0.14 cd	5.96 ± 0.02d	21.73 ± 0.2ab	67.85 ± 0.3a	139.12 ± 14.0a	9.21 ± 0.11a	2.21 ± 0.10a	37.62 ± 4.43bc	9.86 ± 1.09c
W_3_	3.15 ± 0.11bc	6.19 ± 0.10c	21.81 ± 0.4ab	67.46 ± 0.4a	137.94 ± 9.3a	9.15 ± 0.08a	2.18 ± 0.06a	31.91 ± 3.99c	11.29 ± 1.09bc
W_4_	3.41 ± 0.19a	6.69 ± 0.02b	21.34 ± 0.2bc	67.31 ± 0.2a	137.25 ± 11.1a	9.13 ± 0.10a	2.15 ± 0.04a	30.00 ± 5.89c	13.21 ± 1.14b
W_5_	3.22 ± 0.12ab	7.58 ± 0.03a	20.45 ± 0.3d	67.27 ± 0.4a	127.71 ± 15.1ab	9.11 ± 0.03a	2.13 ± 0.02a	29.05 ± 5.14c	15.84 ± 1.45a
CK_1_	2.30 ± 0.09e	7.75 ± 0.18a	20.84 ± 0.4 cd	66.93 ± 0.7a	106.61 ± 5.0b	9.04 ± 0.05a	2.11 ± 0.03a	46.43 ± 1.84a	5.82 ± 1.56d
W_1_	2.95 ± 0.11 cd	5.69 ± 0.46c	21.92 ± 0.3a	67.94 ± 0.9a	142.33 ± 15.4a	9.19 ± 0.04a	2.23 ± 0.05a	36.43 ± 2.98ab	9.46 ± 1.37 cd
W_1_	3.13 ± 0.12c	6.05 ± 0.11bc	21.73 ± 0.3a	67.72 ± 0.5a	135.26 ± 10.3a	9.19 ± 0.10a	2.23 ± 0.03a	33.09 ± 2.17bc	10.95 ± 2.6bcd
W_1_	3.43 ± 0.16b	6.35 ± 0.14b	21.54 ± 0.5a	67.51 ± 0.4a	132.73 ± 8.9a	9.14 ± 0.10a	2.21 ± 0.03a	29.76 ± 3.62 cd	12.76 ± 1.78bc
W_1_	3.82 ± 0.17a	6.45 ± 0.15b	21.47 ± 0.4a	67.41 ± 0.4a	132.55 ± 9.9a	9.12 ± 0.07ab	2.21 ± 0.06a	25.95 ± 4.89d	13.54 ± 1.12b
W_1_	3.56 ± 0.14ab	5.66 ± 0.13c	21.93 ± 0.2a	67.65 ± 0.2a	125.91 ± 3.6ab	9.07 ± 0.05ab	2.17 ± 0.03a	23.57 ± 3.29d	17.48 ± 2.30a
CK_2_	2.71 ± 0.15d	7.03 ± 0.11a	21.28 ± 0.3a	67.16 ± 0.3a	108.07 ± 8.1b	8.99 ± 0.06b	2.16 ± 0.03a	42.38 ± 1.60a	7.63 ± 1.34d
W_11_	2.65 ± 0.11bc	5.49 ± 0.09c	22.15 ± 0.3a	67.93 ± 0.4a	144.62 ± 14.1a	9.23 ± 0.09a	2.23 ± 0.02a	34.05 ± 3.09b	10.12 ± 1.54 cd
W_12_	2.85 ± 0.18b	5.62 ± 0.19c	21.91 ± 0.5a	67.87 ± 0.3a	138.17 ± 9.5a	9.20 ± 0.09ab	2.23 ± 0.06a	27.38 ± 3.02bc	11.49 ± 1.31c
W_13_	3.11 ± 0.10a	6.08 ± 0.21b	21.42 ± 0.2ab	67.88 ± 0.5a	142.33 ± 3.9a	9.21 ± 0.09ab	2.21 ± 0.06a	27.38 ± 4.55c	13.16 ± 1.53bc
W_14_	3.27 ± 0.12a	6.79 ± 0.22a	21.27 ± 0.4b	67.22 ± 0.2a	138.35 ± 6.2a	9.12 ± 0.09ab	2.21 ± 0.03a	25.24 ± 2.05 cd	15.15 ± 1.58ab
W_15_	3.18 ± 0.13a	6.34 ± 0.18b	20.91 ± 0.5b	67.94 ± 0.7a	133.49 ± 6.6a	9.05 ± 0.03bc	2.19 ± 0.05a	20.01 ± 1.79d	17.6 ± 1.41a
CK_3_	2.43 ± 0.12c	6.83 ± 0.15a	20.81 ± 0.3b	67.72 ± 0.3a	113.19 ± 11.4b	8.93 ± 0.09c	2.17 ± 0.06a	39.76 ± 2.58a	8.04 ± 1.92d

Conduct a significance analysis by treating the same pipe spacing as a group. All data are mean±SD; different letters (a, b, c, d, e) indicate significant differences at 0.05 level. Fractional data quoted from“Effects of organic and inorganic fertilizer application on sunflower yield, quality and saline-alkali soil improvement under the subsurface drainage” [31].

According to the correlations between the index values and management measures, palmitic acid showed a downward trend with an increase in the proportion of organic fertilizer application, and the lowest contents of palmitic acid appeared at 100% organic fertilizer nitrogen under the conditions of 10m and 20m pipe spacings. On the contrary, oleic acid content and linoleic acid content showed an upward trend. The soil available nitrogen content, soil available phosphorus content, and soil organic matter content basically increased linearly with an increase in the proportion of organic fertilizer application. However, the evaluation differences of these indicators were not very obvious. With the increase in the proportion of organic fertilizer, the salt reduction efficiency of topsoil salt was significantly improved. Under the three subsurface pipe spacings, the salt reduction rate for the CK treatment was the highest, followed by that for 100% organic fertilizer nitrogen. The highest salt reduction rate was observed for the 10m pipe spacings; The smaller the pipe spacings, the higher the salt reduction rate. N_2_O emissions were lower under no-fertilization treatment. The larger the pipe spacings, the higher the N_2_O emissions; when the distance between pipe spacings was 20m, the emissions were at their maximum.

### Establishment of the entropy–TOPSIS Evaluation model

Evaluating the subsurface drainage and bio-organic fertilizer management model for sunflower growth involves multiple objectives and indicators, and determining the optimal model requires the use of mature statistical tools. Different sunflower planting patterns can produce different yields, qualities, and environmental impacts. For example, some planting methods may obtain high yields but exacerbate soil salinization or emit more N_2_O; some planting methods protect the soil, but their yield and quality decrease. The specific steps of the improved TOPSIS method were as follows:

#### (1) Constructing the evaluation matrix.

Let Rij denote the original matrix of the subsurface drainage and bio-organic fertilizer management model for sunflower growth, containing the original values for treatment j corresponding to index i, where i = 1, 2, …, n and j = 1, 2, …, m. Since nitrogen fertilizer was not applied to treatments CK_10_, CK_15_, and CK_20_, these three treatments were not analyzed in the TOPSIS entropy weight method comprehensive evaluation, and only the exogenous measures were evaluated and analyzed. There were 15 planting modes, with a total of nine indicators for the aspects of yield increase, quality improvement, soil improvement, salt reduction, and emission reduction; therefore, we set n = 9 and m = 15.


$$R_{ij}={\left(R_{ij}\right)}_{n\times m}=\begin{array}{cccc} r_{11} & r_{12} & ... & r_{1m}\\ ... & ... & ... & ...\\ r_{n1} & r_{n2} & ... & r_{nm} \end{array}$$ (4)

#### (2) Standardizing the matrix.

Since the evaluation indexes include positive attribute indexes and negative attribute indexes, it is necessary to standardize the matrix in order to reduce the dimensional coefficient error.


$$\text{Positive Attribute Indexes }R_{ij}^{'}=\frac{R_{ij}-minR_{ij}}{maxR_{ij}-minR_{ij}}$$ (5)

$$\text{Negative Attribute Indexes }R_{ij}^{'}=\frac{maxR_{ij}-R_{ij}}{maxR_{ij}-minR_{ij}}$$ (6)

Here, $R_{\text{ij}}$is the (i,j)th index value from the original matrix; $R_{\text{ij}}^{'}$is the (i,j)th index value after standardization;$\max R_{\text{ij}}$ is the maximum value and $\min R_{\text{ij}}$is the minimum value.

#### (3) Calculating the weight via the entropy weight method.


$$R_{ij}=\frac{R_{ij}^{'}}{\sum_{j=1}^{m}R_{ij}^{'}}$$ (7)

$$e_{i}=\frac{\sum_{j=1}^{m}p_{ij}\text{in }p_{ij}}{\text{in }n}$$ (8)

$$g_{i}=1-e_{i}$$ (9)

$$W_{i}=\frac{g_{i}}{\sum_{j=1}^{n}g_{i}}$$ (10)

Here, $P_{\text{ij}}$is the standardized proportion of each index; ${\text{e}}_{\text{i}}$is the information entropy of each index; ${\text{g}}_{\text{i}}$is the coefficient of difference of each index;$W_{\text{i}}$is the weight value of each index.

#### (4) Constructing the TOPSIS Model.


$$V_{ij}=W_{i}\times R_{ij}$$ (11)

$$V^{+}=\left(V_{1}^{+},V_{2}^{+},\ldots ,V_{m}^{+}\right)=\left\{maxV_{ij} |i=1,2,\cdots n\right\}$$ (12)

$$V^{-}=\left(V_{1}^{-},V_{2}^{-},\ldots ,V_{m}^{-}\right)=\left\{m\text{in}V_{ij} |i=1,2,\cdots n\right\}$$ (13)

$$S_{\text{j}}^{+}=\sqrt{{\sum_{\text{i}=1}^{\text{n}}\left(V_{\text{i}}^{+}-V_{\text{ij}}\right)}^{2}}$$ (14)

$$S_{\text{j}}^{-}=\sqrt{{\sum_{\text{i}=1}^{\text{n}}\left(V_{\text{i}}^{-}-V_{\text{ij}}\right)}^{2}}$$ (15)

$$C_{j}=\frac{S_{j}}{S_{j}^{+}+S_{j}^{-}}$$ (16)

Here,$V_{\text{ij}}$is the weighted matrix calculated by normalizing the weights and matrices; $V^{+}$and$V^{\text{-}}$are the positive and negative ideal solutions of each index in the weighted matrix; $S_{\text{j}}^{+}$and$S_{\text{j}}^{\text{-}}$are the distances to the positive and negative ideal solutions (the optimal distance, the worst distance).$C_{\text{j}}$is the relative posting progress obtained based on the optimal distance and the worst distance; the larger the relative posting progress, the more optimal the mode.

### TOPSIS entropy weight method comprehensive evaluation analysis

(1) According to formula (4) , evaluation matrix R was constructed from the values obtained for the sunflower yield, palmitic acid content, oleic acid content, linoleic acid content, soil available phosphorus content, soil available nitrogen content, soil organic matter content, topsoil salt reduction efficiency, and N_2_O emissions. According to formula (5)  ~  (6) , normalize the matrix to obtain matrix R’.

R9×15'=2.745.7522.1267.73134.939.212.2346.628.852.935.9521.7367.85139.129.212.2137.629.863.156.1921.8167.46137.949.152.1831.9111.293.416.6921.3467.31137.259.132.1530.0013.213.227.5820.4567.27127.719.112.1329.0515.842.955.6921.9267.94142.339.192.2336.439.463.136.0521.7367.72135.269.192.2333.0910.953.436.3521.5467.51132.739.142.2129.7612.763.826.4521.4767.41132.559.122.2125.9513.543.565.6621.9367.65125.919.072.1723.5717.482.655.4922.1567.93144.629.232.2334.0510.122.855.6221.9167.87138.179.202.2327.3811.493.116.0821.4267.88142.339.212.2127.3813.163.276.7921.2767.22138.359.122.2125.2415.153.186.3420.9167.94133.499.052.1920.0117..6

**Fig 1:**
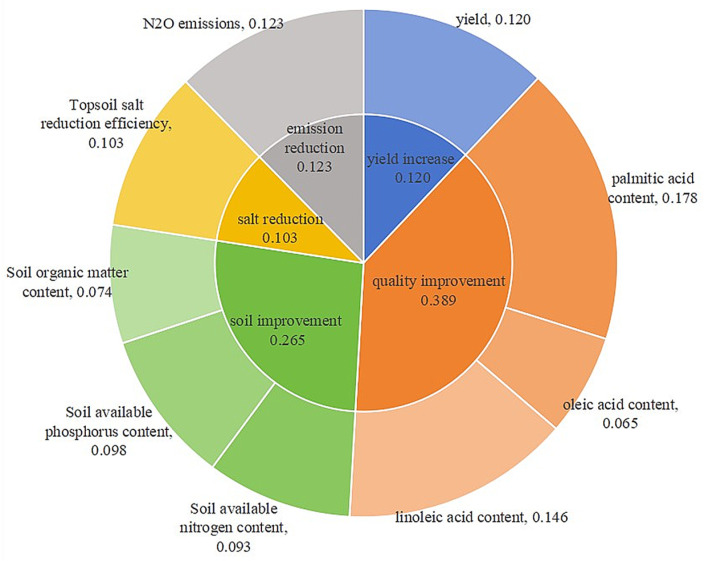
Weighting percentages of the index. (The data is calculated according to formula 7-10).

**Fig 2:**
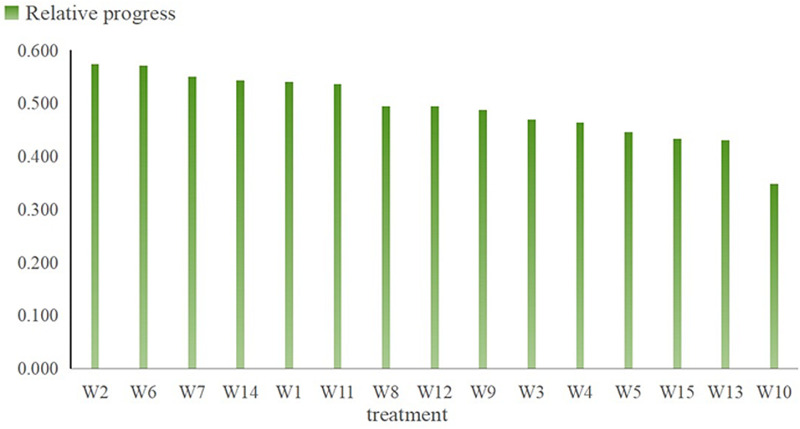
The relative progress ranking of each treatment.

(2) According to formulas(7) ~ (10), calculate the information entropy and weight of the standardized index. The results of the TOPSIS entropy weight method are shown in Fig 1. The weight order was as follows: palmitic acid content>  linoleic acid content >  N_2_O emissions>  yield>  topsoil salt reduction efficiency>  soil available phosphorus content>  soil available nitrogen content>  soil organic matter content>  oleic acid content. The Indicator weight reflects its degree of influence on the comprehensive evaluation.

(3) Formulas (11) ~  (13) were used to calculate the standard matrix to obtain its positive ideal value and negative ideal value.
(4) Calculate the optimal distance, the worst distance, and relative posting progress according to formulas(14) ~ (16), as shown in Table 5. The standardized matrix was sorted and reordered on the basis of the relative posting progress. The greater the relative posting progress, the better the indicated processing effect of this method. Fig 2 illustrates the relative progress ranking of each treatment. The optimal treatment is W_2_, and the worst treatment is W_10_.

**Table 5:** Optimal distance, worst distance, and relative progress of each treatment.

Treatment	Optimal distance	Worst distance	Relative progress
W_1_	0.202	0.238	0.541
W_2_	0.173	0.233	0.574
W_3_	0.194	0.172	0.470
W_4_	0.198	0.171	0.464
W_5_	0.244	0.196	0.446
W_6_	0.188	0.250	0.571
W_7_	0.171	0.209	0.550
W_8_	0.180	0.177	0.495
W_9_	0.196	0.186	0.487
W_10_	0.268	0.143	0.348
W_11_	0.219	0.254	0.537
W_12_	0.214	0.209	0.495
W_13_	0.178	0.211	0.542
W_14_	0.221	0.167	0.430
W_15_	0.238	0.182	0.433

Note: the optimal distance and the worst distance are the distances to the positive and negative ideal solutions according to formulas (11) ~  (15), and the relative posting progress obtained based on the optimal distance and the worst distance according to formulas (16).

(5) The optimal treatment method is a pipe spacing of 10m and organic fertilizer nitrogen 75% and inorganic fertilizer nitrogen 25%. Under this treatment, the relative progress reached 0.574, the yield reached 2.93 t/ha, the oleic acid content reached 21.73%, the soil organic matter content reached 2.21%, the soil salt reduction efficiency reached 37.62%, and N_2_O emissions reached 9.86 kg/ha.

In addition to treatment W_2_ having the best comprehensive benefit, it is not difficult to see that other treatments have their own favorable characteristics. For example, it had the highest yield among all processing methods; the yield reached 3.82 t/ha, but the salt reduction and N_2_O emission reduction were not good: the salt reduction rate and N_2_O emissions were 38.96% and 51.69% lower than the highest values, respectively. The best treatment for soil fertility is W_11_: with pipe spacings of 20m and 100% organic fertilizer nitrogen, the contents of soil available phosphorus, soil available nitrogen, and soil organic matter were 144.62mg/kg, 9.23mg/kg, and 2.23%, respectively. The oleic acid content and linoleic acid content were also the best under this treatment. Salt and N_2_O reduction rates are the best under W_11_ treatment: with 10m pipe spacings and 100% organic fertilizer nitrogen, the soil salt reduction efficiency was 42.62% (113% higher than the lowest value), and N_2_O emissions were 8.85 kg/ha (49% lower than the highest emissions), but the sunflower yield was lower in this treatment mode (28.65% lower than W_9_ treatment).

In summary, the TOPSIS entropy weight method was able to comprehensively evaluate the management mode of subsurface drainage and bio-organic fertilizer application for sunflower growth. The comprehensive evaluation results are as follows: when the pipe spacings are 10m, and the fertilizer application mode is 75% organic fertilizer nitrogen +  25% inorganic fertilizer nitrogen, its processing method is optimal.

## Discussion

**Fig 3:**
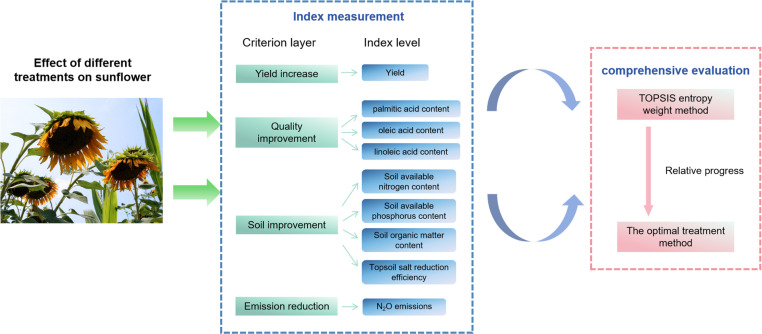
The technical route of collaborative management measures of sunflower subsurface drainage and bio-organic fertilizer.

Sunflower is a cash crop; it has a better improvement effect on saline-alkali land and is of great significance in sustainable agricultural development. This study designed three subsurface drainage spacings and six methods of combined application of organic fertilizer and observed the relevant indexes by means of experiment. In addition, a quantitative evaluation was carried out using the TOPSIS entropy weight method. The technical route is shown in Fig 3.

When the organic fertilizer nitrogen was 25% and the inorganic fertilizer nitrogen was 75%, the yield was the highest. Under the same subsurface pipe spacings, as the proportion of organic fertilizer application increases, the yield shows an initial increase followed by a decrease. This shows that mixing organic and inorganic fertilizers can increase the yield of sunflowers. The research results of He Wang and Liu et al. [32,33]. This indicates that soil fertility is effectively improved with the application of organic and inorganic fertilizers, thereby increasing crop yields. Studies have shown that although sunflower yield can be significantly improved by applying nitrogen fertilizer, sunflower growth can be inhibited by applying too much nitrogen fertilizer, thus reducing the yield [34]. Under the same fertilization conditions, the highest yield of all treatments was achieved with a pipe spacing of 15m. Relevant studies have shown that subsurface drainage creates a better-growing environment for crops [35,36]. With a smaller pipe spacing, there is greater water displacement, reduced soil moisture, and a loss of available nitrogen in the topsoil, reducing the yield [37,38]. Larger subsurface pipe spacings lead to poor drainage and salt retention in the topsoil, which also reduces yield.

The relationship between quality improvement index and pipe spacing is not significant but is affected by the combination of organic and inorganic fertilizers; increasing the proportion of organic fertilizer leads to an increase in oleic acid and linoleic acid content, while palmitic acid decreases. The research results explained this phenomenon: soil fertility increases with the increase of organic fertilizer proportion, further increasing the oleic acid and linoleic acid content of sunflowers, which makes crop growth faster and better [36,39–40].

With the application of organic fertilizer, soil fertility increased. Scholars have discovered through experiments that an increase in the application ratio of organic fertilizer increases the contents of soil available nitrogen, phosphorus, and organic matter [41,42]. The salt reduction rate in soil without nitrogen fertilizer was significantly higher than those under fertilizer treatments, which is due to the introduction of base ions with fertilizer application, reducing the soil salt reduction rate [43]. The salt reduction rate of the topsoil was directly proportional to the amount of organic fertilizer added; the salt reduction rate was the highest with pipe spacings of 10m, significantly higher than those for pipe spacings of 15m and 20m. Experiments in coastal saline-alkali land showed that organic fertilizer can be used to replace high-dose fertilizer to improve soil salinity [44] effectively. Hong Yu [45] showed that the organic fertilizer decreased the pH value of soil in a study of irrigated silt in Ningxia. Scholars have concluded that subsurface drainage can reduce soil salinity [46,47]. This study found that the soil salt reduction rate was the highest when the subsurface drainage pipe spacings were 10m. Zheng Yan [35] conducted an experiment in the Hetao area and set different subsurface pipe spacings during sunflower planting. Through the experiment, it was found that the salt control effect of a 20m pipe spacings was better than that of a 30m pipe spacings. Zhang Li [48] reported that reducing the subsurface spacing can effectively improve drainage and salt discharge. Zhou Liying [49] pointed out that soil water and salt are more easily discharged with a smaller subsurface pipe spacing due to the shortened migration distance.

The more organic fertilizer was applied, the lower the N_2_O emissions, and the emissions from applying nitrogen fertilizer were higher than those not applying nitrogen fertilizer. Organic fertilizer increases the soil organic matter content; this organic matter consumes oxygen during the decomposition process and inhibits the nitrification and mineralization of soil organic nitrogen, thus reducing N_2_O emissions [50,51]. However, there were also studies with the opposite conclusion: organic fertilizers do not promote emissions reduction [52]. The differences in these outcomes may be due to differences in the nitrogen application levels and types of organic fertilizer, which reduce their significance. The emission reduction effect of the smallest pipe spacings was the best due to the high salt reduction rate of the small pipe spacings. Studies have shown that N_2_O emissions are significantly correlated with salt, and salt changes the solubility of N_2_O in soil [53].

A significant analysis of experimental data using SPSS can comprehend the impact of a single index on sunflower growth, but its evaluation is relatively narrow and involves a single index, such as yield or quality improvement, and cannot evaluate all aspects. In an actual sunflower growth evaluation, we should pay more attention to the comprehensive impact of yield increase, quality improvement, soil improvement, salt reduction, and emission reduction. In this study, the TOPSIS entropy weight method was used to evaluate a management model of sunflower subsurface drainage and bio-organic fertilizer application. This method reduced the influence of perceived factors and was more objective. The conclusion of this evaluation is credible. As an evaluation method, the TOPSIS entropy weight method is comprehensive and adaptable, and it can play a guiding role in the selection of crop production methods [54,55].

## Conclusions

Our overall results showed that under consistent subsurface drainage conditions, the yield showed an initial rise followed by a decline with the increase of organic fertilizer, the palmitic acid content showed a downward trend, while the oleic acid content and linoleic acid content exhibited a rising trend, and the indexes of soil improvement displayed an upward trend. When nitrogen fertilizer was not applied, the salt reduction rate was higher. Furthermore, increasing the combined application ratio of organic fertilizer improved the soil salt reduction rate and reduced N_2_O emissions. Under uniform fertilization conditions, the yield was highest when the pipe spacings were 15m; however, no significant relationship was observed between the quality improvement index or soil improvement index and pipe spacing. Sunflower subsurface drainage was a useful way to control soil salt content and improve soil salinization. This study revealed that under hidden pipe condition S1, both the soil salt reduction rate and emission reduction effect were at their peak. According to the TOPSIS entropy weight method evaluation results, the optimal application mode involved 75% organic fertilizer nitrogen +  25% inorganic fertilizer nitrogen with 10m pipe spacings. During the cultivation of sunflowers in saline-alkali areas, this conclusion can provide some theoretical basis; however, further assessment is required to evaluate actual growth in conjunction with economic and social benefits, environmental factors, etc.
